# A systems perspective on placental amino acid transport

**DOI:** 10.1113/JP274883

**Published:** 2018-09-07

**Authors:** Jane K. Cleal, Emma M. Lofthouse, Bram G. Sengers, Rohan M. Lewis

**Affiliations:** ^1^ Faculty of Medicine University of Southampton Southampton UK; ^2^ Institute of Life Sciences University of Southampton Southampton UK; ^3^ Faculty of Engineering and the Environment University of Southampton Southampton UK

**Keywords:** trans-epithelial transport, fetal growth restriction, computational modelling

## Abstract

Placental amino acid transfer is a complex process that is essential for fetal development. Impaired amino acid transfer causes fetal growth restriction, which may have lifelong health consequences. Transepithelial transfer of amino acids across the placental syncytiotrophoblast requires accumulative, exchange and facilitated transporters on the apical and basal membranes to work in concert. However, transporters alone do not determine amino acid transfer and factors that affect substrate availability, such as blood flow and metabolism, may also become rate‐limiting for transfer. In order to determine the rate‐limiting processes, it is necessary to take a systems approach which recognises the interdependence of these processes. New technologies have the potential to deliver targeted interventions to the placenta and help poorly growing fetuses. While many factors are necessary for amino acid transfer, novel therapies need to target the rate‐limiting factors if they are going to be effective. This review will outline the factors which determine amino acid transfer and describe how they become interdependent. It will also highlight the role of computational modelling as a tool to understand this process.

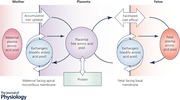

## Introduction

Placental transfer provides the amino acids required for fetal growth and metabolism. The fetus requires amino acids for protein accretion, metabolic processes and biosynthetic pathways but can only obtain these from the placenta. Understanding the process of placental transfer is important as it provides the amino acids required for appropriate growth and development of the fetus. Indeed, impaired placental amino acid transfer is associated with reduced fetal growth, which is itself associated with complications in the perinatal period and elevated rates of chronic disease in later life. To improve outcomes from fetal growth restriction (FGR), we need to appreciate how placental amino acid transfer operates as an integrated system. This integrated understanding will allow targeting of the right mechanisms to prevent or treat impaired placental amino acid transfer.

The human placenta mediates net transfer of amino acids to the fetus, with amino acid concentrations being higher in fetal plasma compared to maternal plasma, indicating an active transfer process across the placenta (Cetin *et al*. 1996). The most notable exception to this is the amino acid glutamate for which there is net placental uptake from the fetus. Amino acid transport across the placenta is a complex process that is determined by multiple interacting factors. These include transporter characteristics, placental structure, maternal and fetal blood flow and the amino acid concentrations within the maternal, placental and fetal compartments.

Multiple amino acid transporters are differentially expressed in the maternal facing microvillous (MVM) and fetal facing basal membranes (BM) of the placental syncytiotrophoblast. Transporter activity is not simply determined by the protein expression levels, but also by the factors that control substrate levels on both sides of the membrane (Panitchob *et al*. 2016). The factors which affect amino acid levels include blood flow, metabolism and the action of multiple amino acid transporters. The trafficking of amino acid transporter protein to the plasma membrane is also an important factor in the regulation of amino acid transfer (Chen *et al*. 2015, 2017). Any effective intervention needs to target the rate‐limiting factors in the system, but the interaction and interdependency between these factors makes identifying the rate‐limiting factors difficult.

Placental amino acid transfer, must be thought of as a system rather than simply focusing on individual mechanisms (Lofthouse *et al*. 2015). A computational modelling approach is helping us to predict the rate‐limiting factors in this system so that these can be the focus of future intervention‐based studies (Panitchob *et al*. 2015, 2016; Lofthouse *et al*. 2016). Placental amino acid transfer can be measured experimentally in the perfused placenta (Cleal *et al*. 2011) and uptake studies can be performed in villous fragments or purified trophoblast preparations (Rosario *et al*. 2013; Ditchfield *et al*. 2015). The isolation of purified MVM and BM vesicles can also be used to study transporters in these membranes in isolation (Speake *et al*. 2003; Panitchob *et al*. 2015). This review will provide an overview of the current knowledge of amino acid transfer across the human placenta. We will discuss the mechanisms of amino acid transfer, how these interact and how computational modelling can be used to identify rate‐limiting steps. Identifying the rate‐limiting steps is crucial if clinical interventions aimed at improving placental transfer and fetal growth are to be successful.

### Computational modelling of amino acid transfer

The complex and interdependent nature of the interactions underlying placental amino acid transfer mean it is hard to identify the rate‐limiting processes. Computational modelling provides a tool to interrogate the complex activity of the system. Modelling is most useful when it is based on experimental data and makes predictions that can be tested in future experiments. The typical model parameters of a compartmental model are outlined in Table 1. Crucial data for placental modelling include both maternal and fetal venous‐arterial differences and tissue levels; otherwise mass balance cannot be validated. An assumption of compartmental models is that the compartments are well mixed and it is necessary to consider the time scales over which the relevant processes occur. We assume transporter behaviour follows the principles of carrier‐mediated models, but phenomenological transport models can be applied depending on the data available (Widdows *et al*. 2015). Placental perfusions provide the best system for modelling transfer and the combination of perfusions with modelling has provided unexpected insight into placental amino acid, fatty acid and cortisol transport (Lofthouse *et al*. 2015; Panitchob *et al*. 2016; Perazzolo *et al*. 2017a; Stirrat *et al*. 2018). Recent work modelling oxygen transfer has recently been reviewed elsewhere (Nye *et al*. 2018). This combined modelling and experimental approach can also be applied to transporter function and in combination with experiments in MVM membrane vesicles has demonstrated that the LAT2 transporter is not an obligate exchanger (Panitchob *et al*. 2015; Widdows *et al*. 2015).

**Table 1:** Typical parameters for a compartmental model of placental transfer in the perfused placenta

Parameters	Compartments	Source from which values are typically determined
Compartment volume	Intervillous space; placenta; fetal capillary volume	Literature or experimentally determined
Flow rates	Maternal arterial; fetal arterial	Experimental design
Initial concentration (for each substrate)	Maternal intervillous space; fetal capillary	Equal to initial perfusion buffer
Arterial input concentration (for each substrate)	Maternal artery; fetal artery	Experimental design
Membrane transport: diffusion or transporter model, substrate specific	Maternal ↔ placental; placental ↔ fetal	Determined by the model based on experimental data
Metabolic rate	Placental tissue	Predicted by the model based on experimental data
Paracellular diffusion	Maternal ←→ fetal	Experimental measurements, e.g. creatinine transfer

These approaches allow us to test our understanding of the physiology underlying placental amino acid transfer. In doing so, we will be able to more accurately predict those factors that are likely to be rate‐limiting and those factors that are not. Understanding which factors (e.g. structure, blood flow, transporter expression, etc.) are likely to be rate‐limiting will help focus future research and clinical interventions.

A strength of modelling is that when used in combination with experimental data it allows our biological understanding to be tested. If a model is able to fit experimental data across a range of different experimental conditions then it is likely that the assumptions underlying it are reasonable. A model that is not able to fit the experimental data is still informative as it suggests that the underlying assumptions are incorrect or at least incomplete. This was the case in our work on amino acid and lipid transport where initial models failed because they did not include metabolism (Lofthouse *et al*. 2016; Perazzolo *et al*. 2017a). The limitation of models comes when they are not closely linked to experimental data or where modelling is based on a limited set of experimental data. Models are also limited where there are too many unknown parameters, which cannot be uniquely determined by the model. For this reason, it is better to start with simple models and test them thoroughly before building on these.

Current mechanistic models of placental transfer do not reflect how hormonal or feedback regulation may affect the system. As regulatory pathways become better defined these too can be modelled and integrated with mechanistic models. Modelling could also address other interactions, such as the way in which spatial differences in oxygen tension within the lobule may affect energy metabolism and so amino acid transfer. Ultimately, the aim should be to develop a virtual placenta as part of a virtual mother and fetus.

### Placental structure and blood flow

**Figure 1:**
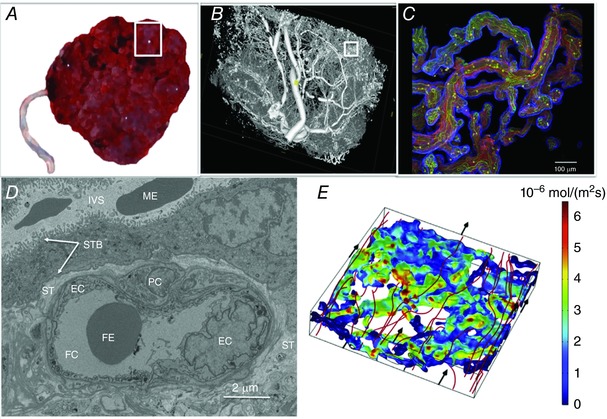
The processes determining placental amino acid transfer operate across a range of scales Amino acids within maternal blood mix effectively between the placental villi before being transported across the apical and basal membranes of the syncytiotrophoblast, diffusing through the stroma and then through the junctions between endothelial cells. *A*, a whole placenta approximately 20 cm across. The white box indicates one placental lobule (∼3 cm across). *B*, micro‐computed tomography image of the region shown in the white box in *A*, illustrating the extent and complexity of the fetal vasculature. The white box in *B* indicates a region of placental villi. *C*, the region shown by the white box in *B* shown as a projection of a whole mount confocal image stack, with the trophoblast stained blue, connective tissue red and endothelium green. *D*, an electron microscopy image of a cross section of a terminal villi. *E*, computational 3D simulation results modelling streamlines of maternal blood flow (m s^−1^) through the intervillous space surrounding placental villi, with streamlines in red depicting the main flow routes (flow direction indicated by black arrows). Colour on the villous surface represents uptake of substrate in that area with red indicating highest solute flux through the villous barrier (mol m^−2^ s^−1^); predicted using the computational model. EC, endothelial cell; FC, fetal capillary; FE, fetal erythrocyte; IVS, intervillous space; ME, maternal erythrocyte; PC, pericyte; ST, villous stroma; STB, syncytiotrophoblast (the microvillous membrane and basal plasma membranes of the syncytiotrophoblast are indicted by the white arrows).

The placenta has a complex structure and specific structural features may be particularly important for the efficiency of amino acid transport (Fig. 1). These features include those affecting blood flow and mixing of amino acids in the blood within the intervillous spaces of the placenta, the surface area available for exchange, the distance over which diffusion occurs and paracellular leak back to the mother. Although these features will affect solute transfer generally, they must have a specific effect on amino acids for them to be rate‐limiting to amino acid transfer.

The size of the placenta and more importantly the surface area of the MVM, which mediates the uptake of amino acids from the maternal circulation, is a key consideration. Classic studies in the P0 knockout mouse suggest that a smaller placenta may become rate‐limiting for amino acid transfer (Constancia *et al*. 2002). In the P0 mouse model, a smaller placenta was initially compensated for by increasing placental transporter activity to maintain fetal weight. However, as gestation progressed fetal growth restriction was observed despite transporter upregulation, indicating that at a certain threshold transporter regulation can no longer compensate for reduced placental size (Sferruzzi‐Perri *et al*. 2011). A decrease in the number and surface area of terminal villi and capillaries has also been reported in placentas from intrauterine growth restricted pregnancies (Mayhew *et al*. 2003).

The interaction between structure and the mixing of maternal blood within the placental intervillous space could also affect amino acid transfer. Low blood flow, or inefficient mixing, can cause regions of low substrate concentration to form in the intervillous space (Perazzolo *et al*. 2017b). Amino acid transfer may be susceptible to inefficient mixing and if substrate gradients are not maintained, exchange transporters may mediate reverse transport of specific amino acids (Panitchob *et al*. 2016). The effect of maternal and fetal blood flow on placental phenylalanine transfer has been studied in the isolated perfused placenta (Lofthouse *et al*. 2016). Neither maternal nor fetal flow was found to be limiting for amino acid transfer within the range of flows tested. However, at very low substrate concentrations maternal flow was demonstrated to be limiting for amino acid uptake (though not transfer). So, while flow does not appear to be limiting for placental amino acid transfer within the physiological range, this study illustrates interdependence between flow and substrate availability.

Diffusion through the stoma or endothelium is unlikely to be rate‐limiting to amino acid transfer as perfusion data indicate that movement of solute from the fetal circulation to the BM and back occurs rapidly (Lofthouse *et al*. 2016). There is limited evidence that transplacental amino acid transfer is also rapid (Sengers *et al*. 2010). As such, we do not believe that stromal diffusion or the endothelium is likely to be limiting for amino acid transfer.

Amino acid transfer is susceptible to the effects of paracellular transfer as amino acid concentrations are higher in the fetal circulation, and so net diffusion would be back to the maternal circulation. There is significant paracellular diffusion of small solutes, including amino acids, across the placenta but the pathway by which this occurs is unclear. While rates of paracellular diffusion are fairly constant in normal placentas, this may vary in disease states, and paracellular diffusion is correlated to area of syncytial damage (Brownbill *et al*. 2000).

### Placental amino acid transport

All of the amino acid transporters in the placenta are members of the SLC superfamily (Table 2; Hediger *et al*. 2013). The expression of these transporters at RNA and protein levels is becoming clear with next generation sequencing and proteomic approaches. However, whether specific amino acid transporters are localised in the MVM, the BM or other placental cell types often remains uncertain, with differences reported between immunological and functional localisation (Cleal *et al*. 2011; Widdows *et al*. 2015).

**Table 2:** Amino acid transporter systems in the placenta

Human gene name (protein/system)	Expression and localisation of mRNA expression from Simner *et al*. (2017)	Mechanism	Substrates
*SLC1A1* (EAAT3/X^AG^) *SLC1A2* (EAAT2/X^AG^) *SLC1A3* (EAAT1/X^AG^) *SLC1A6* (EAAT4/X^AG^) *SLC1A3* (EAAT5/X^AG^)	mRNA; activity on MVM and BM (cannot distinguish family member); SLCA1,2,3 protein expressed but location unclear (Moe & Smith, 1989; Hoeltzli *et al*. 1990; Noorlander *et al*. 2004)	3Na^+^/H^+^/AA cotransport/K^+^ exchange	D, E (Kanai *et al*. 2013)
*SLC1A4* (ASCT1/ASC)			A, S, C, T
	mRNA; activity on BM (Cleal *et al*. 2011)	Na^+^ dependant exchanger	
*SLC1A5* (ASCT2/ASC)			A, C, Q, S, T, N (Kanai *et al*. 2013)
*SLC3A1* (rBAT) *SLC3A2* (4F2hc)	mRNA mRNA; protein on MVM and BM (Palacin & Kanai, 2004)		Chaperone subunit for specific SLC7 transporters
*SLC6A6* (TAUT)	mRNA; activity and protein on MVM (Roos *et al*. 2004; Desforges *et al*. 2013)	Na^+^/Cl^−^ dependant cotransporter	Taurine (Pramod *et al*. 2013)
*SLC7A1* (CAT1/y^+^) *SLC7A2* (CAT2B/y^+^) *SLC7A3P* (CAT3/y^+^)	mRNA; activity on MVM and BM; CAT1 protein on BM (Speake *et al*. 2003).	Electrogenic uniporter	R, H, K (Fotiadis *et al*. 2013)
*SLC7A5* (LAT1/L) *SLC7A8* (LAT2/L)	mRNA; LAT2 activity on MVM and LAT1 activity on BM; both proteins on MVM and BM (Cleal *et al*. 2011; Widdows *et al*. 2015)	Exchanger; requires 4F2hc (SLC3A2)	F, Y, W, M, V, I, L, H, BCH L, A, S, T, C, F, Y, W, BCH, N, H, I, M, V, Q, G (Fotiadis *et al*. 2013)
*SLC7A7* (y^+^LAT1/y^+^L) *SLC7A6* (y^+^LAT2/y^+^L)	mRNA; activity on MVM and BM but cannot distinguish family member (Ayuk *et al*. 2000; Cleal *et al*. 2011)	Exchanger; requires 4F2hc (Na^+^ dependant for neutral amino acids)	R, H, K, M, ^A^L (Fotiadis *et al*. 2013)
*SLC7A9* (b^0,+^AT)	mRNA; activity inconclusive.	Requires rBAT (SLC3A1)	R, H, K, F, Y, W, T, M, V, I, L (Fotiadis *et al*. 2013)
*SLC7A10* (asc1/asc)	mRNA; no activity on BM (Cleal *et al*. 2011)	Exchanger; requires 4F2hc (SLC3A2)	G, A, S, T, C (Fotiadis *et al*. 2013)
*SLC7A11* (xCT/Xc‐)	mRNA	Exchanger; requires 4F2hc (SLC3A2)	Cystine, E (Fotiadis *et al*. 2013)
*SLC16A10* (TAT1)	mRNA; activity and protein on BM (Cleal *et al*. 2011)	Facilitated diffusion	F, W, Y A, L (Ramadan *et al*. 2006)
*SLC38A1* (SNAT1/A) *SLC38A*2 (SNAT2/A) *SLC38A4* (SNAT4/A)	mRNA; activity and protein on MVM (Desforges *et al*. 2009)	Na^+^/AA cotransporter; Na/AA cotransport, H antiport	Q, A, N, C, H, S A, N, C, Q, G, H, M, P, S A, N, C, G, S, T (Schioth *et al*. 2013)
*SLC38A3* (SNAT3/N)	mRNA	Na/AA cotransport, H antiport	Q, H, A, N
*SLC38A5* (SNAT5/N)	mRNA; activity and protein on MVM (Day *et al*. 2013)		Q, H, N, S (Schioth *et al*. 2013)
*SLC43A1* (LAT3) *SLC43A2* (LAT4)	mRNA; activity and protein on BM (Cleal *et al*. 2011)	Facilitated diffusion	L, I, V, F, M, BCH (Bodoy *et al*. 2013)

BCH, 2‐aminobicyclo‐(2,2,1)‐heptane‐2‐carboxylic acid. l‐Alanine (A), l‐arginine (R), l‐asparagine (N), l‐aspartate (D), l‐cysteine (C), l‐glutamate (E), l‐glutamine (Q), glycine (G), l‐histidine (H), l‐isoleucine (I), l‐leucine (L), l‐lysine (K), l‐methionine (M), l‐phenylalanine (F), l‐proline (P), l‐serine (S), l‐threonine (T), l‐tryptophan (W), l‐tyrosine (Y), l‐valine (V). **^A^**L: y^+^L, influx but not efflux of l‐Leucine (Chillaron et al.).

Amino acid transporters are divided into three functional classes based on their mode of operation: accumulative, exchange and facilitated transporters (Cleal *et al*. 2011). In order to transport the full range of amino acids to the fetus, the placenta requires the different classes of amino acid transporter to work together on both the MVM and BM of the syncytiotrophoblast. To understand how transporters work together to function as a system, it is important to understand how the activities of the different classes complement each other on the MVM and the BM.

**Figure 2:**
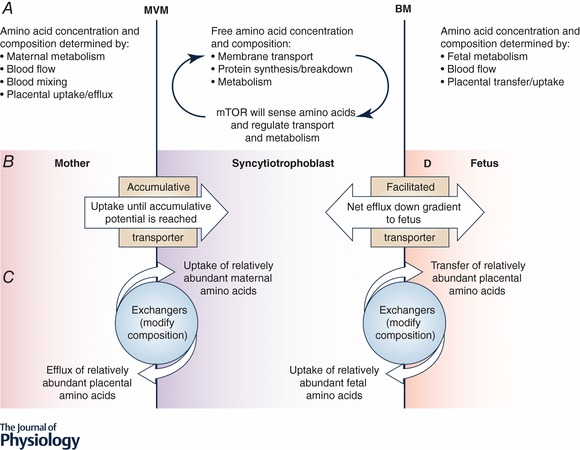
A cartoon showing the location of transporter classes within the MVM and BM of the placental syncytiotrophoblast, and the factors that determine amino acid transfer *A*, maternal, placental and fetal metabolism, blood flow and transport control the gradients which determine transporter activity. *B*, accumulative transporters mediate uptake until their accumulative potential is reached. Functionally this means their activity is affected by low extracellular concentrations and that higher extracellular concentrations above the *V*_max_ will no longer increase uptake. *C*, exchangers will mediate net influx of abundant external substrates in exchange for efflux of relatively higher abundance intracellular substrates. This means that transfer of one substrate will decrease the levels of another. Within physiological limits this will be independent of concentration. *D*, facilitated transporters on the BM will mediate efflux of substrates down the concentration gradient built up by other transporters. Fetal consumption will increase the concentration gradient and increase transfer to the fetus.

Accumulative transporters such as SLC1 and SLC7 (Table 2) mediate net uptake of specific amino acids across the MVM into the syncytiotrophoblast. This creates transmembrane amino acid gradients across the MVM which drive the uptake of other extracellular amino acids via amino acid exchange transporters (Fig. 2). The exchange transporters on the MVM include the SLC7 family and they swap intracellular amino acids for other exchange transporter specific amino acids in the maternal plasma (Fig. 2). Therefore, amino acid exchangers alter the composition (i.e. concentration fractions) of their amino acids substrates within the placenta, but not the overall quantity of amino acids. Neither the accumulative transporters nor the exchangers alone can provide the placenta with all the amino acids it requires, and they must work together to achieve this.

On the BM of the syncytiotrophoblast, the facilitated transporters (SLC16A10 and SLC43; Table 2) mediate net efflux of specific amino acids across the BM into the fetal circulation down their concentration gradient (Cleal *et al*. 2011). These amino acids can be exchanged for other amino acids via exchangers to ensure all the necessary amino acids are transferred to the fetus (Fig. 2). Placental perfusion experiments indicate a pattern of amino acid exchange consistent with Systems ASC and L, but not Systems asc and b^0,+^AT activity on the BM (Cleal *et al*. 2011).

Accumulative transporters, including members of the SLC1 and SCL38 families, are also present on the BM and will mediate uptake of fetal amino acids into the placenta (Hoeltzli *et al*. 1990; Desforges *et al*. 2006). The glutamate and aspartate transporting SLC1s are highly active on the fetal BM and mediate net uptake of fetal glutamate into the placenta where it is metabolised and can act as a counter ion for organic anion exchangers (Day *et al*. 2013; Lofthouse *et al*. 2015). The physiological role of other accumulative transporters on the BM, such as SLC38 family members, is unclear (Desforges *et al*. 2006).

In terms of amino acid transporters working together as a system, we need to understand when membrane transport will become rate‐limiting for amino acid transfer across the placenta. Whether membrane transport is rate‐limiting will depend on the amount of transporter, its mechanism of action and the free amino acid concentrations on both sides of the membrane (Fig. 2).

Accumulative amino acid transporters are secondary active transporters driven by previously established chemical and electrical gradients. The Na^+^, K^+^ and electrical gradients driving uptake are maintained by the Na^+^/K^+^ ATPase, and the H^+^ gradient is also important for specific transporters (Table 2). These electrochemical potential gradients determine how much substrate can be accumulated within the cell. Accumulation of amino acids creates an outwardly directed gradient and influx will only continue while the inward force of the driving electrochemical gradient is greater than that of the substrate being accumulated within the cell. It should be noted that in a physiological system the balance of activity between uptake and efflux means it is unlikely that the system will reach these limits at steady state (Panitchob *et al*. 2016). Membrane transport via accumulative transporters will become rate‐limiting when the driving gradients are depleted or there is just too little transporter in the membrane relative to other transporter classes.

Exchange transporters transport one solute into the cell in exchange for another leaving the cell. Amino acid exchangers are regarded as being obligate exchangers where influx of extracellular amino acids is always coupled to efflux of intracellular amino acids. However, we have recently demonstrated that the exchange transporter LAT2 does not function exclusively as an obligate exchanger (Widdows *et al*. 2015). These transporters mediate net transport until the relative composition (i.e. concentration fractions) of their substrates are the same inside and outside the cell (Fig. 2). This means that at equilibrium, if serine makes up 20% of all the substrates of a transporter outside the cell it will also make up 20% of all these substrates inside the cell regardless of any concentration differences between these pools. The exchangers will mediate a degree of futile cycling, whereby an amino acid is swapped for the same amino acid creating no overall change.

Facilitated transporters mediate transport of solute in both directions across the membrane; net transport will occur down the concentration gradient until an equal concentration of all substrates is achieved on both sides of the membrane. Facilitated transporters become limiting where insufficient transporter is expressed or the transmembrane gradient is reduced. For individual amino acids, too much of a competing substrate could also become limiting.

**Figure 3:**
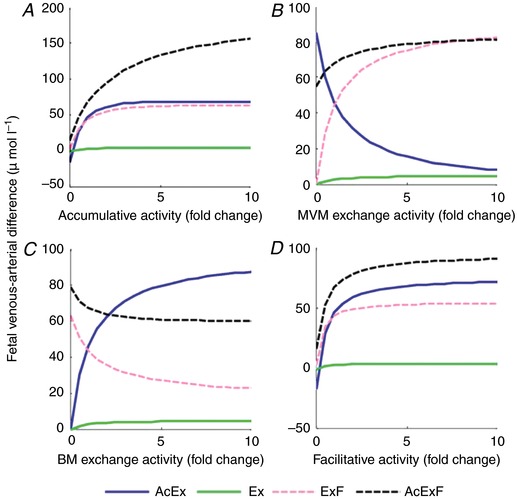
The effect of changing the activity of accumulative (*A*), MVM exchange (*B*), BM exchange (*C*) and facilitative (*D*) transporters on amino acid transfer to the fetus (fetal venous–arterial difference) For simplicity amino acids have been grouped by the classes of transporter which transport them: those transported by accumulative and exchange transporters (AcEx), those transported by exchangers only (Ex), those transported by exchange and facilitated transporters (AcF) and those substrates transported by all three classes of transporter (AcExF). Results represent the sum of all amino acids in each group. Note how in many cases increasing transporter activity has little effect on overall transfer and how increasing exchanger activity can decrease transfer of some substrates (Panitchob *et al*. 2016).

Modelling of the interactions between the different transporter classes in the placenta is therefore needed, and indeed demonstrates the likely complexity of this system. From this work it is clear that simply increasing or decreasing the activity of one transporter cannot always be expected to have a corresponding effect on overall transfer (Panitchob *et al*. 2016). Indeed there are examples, particularly with exchangers, where increased activity of a transporter will decrease the transfer of its substrates (Fig. 3). Changing the activity of a transporter will have different effects on different substrates depending on what other transporters also transport each substrate. This model also makes clear that increasing the activity of a transporter has no effect on transfer if another transporter is limiting. An experimental example of these complex interactions may come from a study where SCL38 transporters were inhibited *in vivo* in rat pregnancy (Cramer *et al*. 2002). Inhibition of SCL38 transporters was shown to alter the activity of SLC1 transporters despite the fact that they do not share any substrates.

It should also be noted that the measured *K*_M_ of a transporter is not a constant but is an apparent *K*_M_ and dependent on the transmembrane substrate gradient (Panitchob *et al*. 2015). For this reason differences in the reported apparent *K*_M_ in different systems, for instance cells and membrane vesicles, should be expected due to the differences in intracellular substrate concentration.

### Metabolism and placental amino acid transfer

As we outline above, amino acid transporter activity depends on the total concentration of amino acids available for transport and on their relative concentrations. Metabolism can therefore influence placental amino acid transfer through its effects on both total and relative concentrations. In addition to metabolism within the placenta, maternal and fetal metabolism will influence arterial plasma amino acid concentrations and so the gradients which determine uptake and efflux. For example, fetal amino acid consumption will increase the maternal–fetal gradient, potentially coupling fetal demand to placental delivery. Here we will focus on placental protein synthesis, breakdown and amino acid interconversion. The use of amino acids as precursors of biosynthetic pathways may also be relevant but is less well understood (Bonnin & Levitt, 2011).

Placental protein synthesis will reduce the pool of amino acids available for transport while the breakdown of protein will increase their availability. In a recent study, we provide evidence that protein synthesis is likely to be a major determinant of placental amino acid transfer (Lofthouse *et al*. 2016). This study suggests that phenylalanine taken up by the placenta is not all available for transfer to the fetus because it has been converted to protein. The effect of metabolism was such that it was not possible to fit a mathematical model to the data unless metabolism was also modelled. Amino acids that enter the placental protein pool are not lost to the fetus but will not be available until that protein is recycled. This delay while amino acids are held within the placental protein pool has implications for the interpretation of tracer studies using amino acids (as opposed to non‐metabolisable amino acid analogues). For amino acids the rate of transfer will be underestimated until the tracer has equilibrated within all metabolic pools, including protein.

Amino acid oxidation within the placenta is coupled to interconversion, preventing the release of toxic ammonia by forming a new amino acid such as alanine or glutamine. As such, oxidation will not significantly reduce overall amino acid concentrations but will change the amino acid composition and thus the gradients which drive their transfer. The human placenta makes glutamine from substrates including glutamate and leucine, and this glutamine is primarily released into the maternal circulation (Day *et al*. 2013). As glutamine is a substrate of many placental amino acid exchangers, the synthesis of glutamine will create gradients that drive uptake of other extracellular amino acids (Day *et al*. 2013).

Amino acid metabolism, and its regulation, have the potential to be rate‐limiting determinants of placental amino acid transfer and so need to be considered when seeking to understand the basis of fetal growth restriction.

### Regulation of placental amino acid transfer

Understanding the regulation of placental amino acid transfer is the key to designing effective intervention strategies to modulate fetal growth. Many studies report stimuli that can regulate specific mechanisms underlying amino acid transfer such as transport and metabolism. However, as we outline in this review the way in which these mechanisms interact is complex and it is only when rate‐limiting processes are targeted that amino acid transfer will be affected.

Regulation of placental amino acid transfer is likely to be based on amino acid sensing and sensing of overall metabolic state. The mechanistic target of rapamycin (mTOR) pathway is central to the regulation of protein metabolism and amino acid transporter activity and is likely to be a key central regulator of placental amino acid transfer (Goberdhan *et al*. 2016). Specifically, intracellular leucine levels are sensed by binding to Sestrin 2 which activates the GATOR2 complex and activates the mTOR complex 1 (mTORC1) pathway (Wolfson *et al*. 2016). mTORC1, which is downregulated in intrauterine growth restriction (Roos *et al*. 2005), modifies plasma membrane trafficking of System A and System L members, suggesting that mTOR regulates fetal growth by modulating specific placental amino acid transporters (Rosario *et al*. 2013).

Regulation of amino acid transporters in the placenta has been demonstrated in response to nutrients and hormones. For example, the MVM expresses receptors for leptin, insulin‐like growth factor‐1 and insulin, which are factors that stimulate System A activity (von Versen‐Hoynck *et al*. 2009; Jones *et al*. 2010). The gene expression of specific placental amino acid transporters can be regulated by maternal factors such as diet, smoking and vitamin D levels (Cleal *et al*. 2015; Day *et al*. 2015; Chen *et al*. 2017). Epigenetic regulation may also be involved in the regulation of amino acid transporter expression in the placenta (Simner *et al*. 2017).

The interrelationships between the determinants of transfer of an amino acid have implications for our understanding of its regulation. A regulatory stimulus that just affects one transporter or metabolic process is unlikely to have a significant effect on the system as a whole unless that target of regulation is the rate‐limiting factor in the system. We suggest that regulatory stimuli will be more effective where they affect the activity of a range of mechanistic targets to up‐ or down‐regulate amino acid transfer.

### Novel therapies, health implications and a systems biology approach

An appreciation of how placental amino acid transfer operates as an integrated system will allow us to target the right mechanisms to prevent or treat impaired placental amino acid transfer and therefore improve outcomes from poor fetal growth. Reduced placental amino acid transfer or transporter activity is associated with reduced fetal growth *in vivo* (Cramer *et al*. 2002). Studies in rodents suggest that decreased amino acid transfer precedes, and may therefore cause, growth restriction (Jansson *et al*. 2006). In humans pregnancies with FGR there is generally decreased transporter activity, and decreased transfer of specific amino acids has been demonstrated using tracers (Paolini *et al*. 2001).

Technologies are being developed which deliver targeted drug or gene therapy to the placenta (King *et al*. 2016; Beards *et al*. 2017; David, 2017). These new approaches have great promise, although promising interventions have proved ineffective in the past (Poston *et al*. 2011). To be effective interventions need to target the aspects of placental function that are rate‐limiting for fetal growth; increasing maternal amino acid levels will only be effective if maternal amino acid levels are an initial problem (Brown *et al*. 2011). Identifying the rate‐limiting processes within the placenta is not always obvious, and computational modelling may provide key insights here. As there may be multiple causes of placental dysfunction, a personalised medicine approach may be necessary with a need to better characterise placental phenotypes and identify accessible biomarkers for this. Just as improving fetal growth may have lifelong benefits, an ill‐judged intervention could cause lifelong detriment (Hanson & Gluckman, 2014).

### Conclusion

Amino acid transfer across the placenta is a complex process which is crucial for normal fetal growth and to create an intrauterine environment which predisposes to lifelong health. In poorly growing fetuses, intervening to improve placental amino acid transfer may improve the intrauterine environment and postnatal health. However, the complex interactions between the different mechanisms mediating amino acid transfer mean we must be careful that any interventions do not have unintended consequences. This will require us to understand the placenta as a system rather than simply focusing on individual mechanisms. The complexity of placental amino acid transfer as a system makes this hard to do at an intuitive level. However, the use of computational modelling can provide a mechanism by which this can be achieved.

## Additional information

### Competing interests

There are no competing interests.

### Author contributions

All authors contributed to the writing of this review. All authors have read and approved the final version of this manuscript and agree to be accountable for all aspects of the work in ensuring that questions related to the accuracy or integrity of any part of the work are appropriately investigated and resolved. All persons designated as authors qualify for authorship, and all those who qualify for authorship are listed.

### Funding

The authors are supported by BBSRC grants BB/L020823/1 and BB/R002762/1.
